# Predicting active-layer soil thickness using topographic variables at a small watershed scale

**DOI:** 10.1371/journal.pone.0183742

**Published:** 2017-09-06

**Authors:** Aidi Li, Xing Tan, Wei Wu, Hongbin Liu, Jie Zhu

**Affiliations:** 1 College of Resources and Environment, Southwest University, Chongqing, China; 2 Chongqing Land Resources and Housing Surveying and Planning Institute, Chongqing, China; 3 College of Computer and Information Science, Southwest University, Chongqing, China; Universidade Federal de Uberlandia, BRAZIL

## Abstract

Knowledge about the spatial distribution of active-layer (AL) soil thickness is indispensable for ecological modeling, precision agriculture, and land resource management. However, it is difficult to obtain the details on AL soil thickness by using conventional soil survey method. In this research, the objective is to investigate the possibility and accuracy of mapping the spatial distribution of AL soil thickness through random forest (RF) model by using terrain variables at a small watershed scale. A total of 1113 soil samples collected from the slope fields were randomly divided into calibration (770 soil samples) and validation (343 soil samples) sets. Seven terrain variables including elevation, aspect, relative slope position, valley depth, flow path length, slope height, and topographic wetness index were derived from a digital elevation map (30 m). The RF model was compared with multiple linear regression (MLR), geographically weighted regression (GWR) and support vector machines (SVM) approaches based on the validation set. Model performance was evaluated by precision criteria of mean error (ME), mean absolute error (MAE), root mean square error (RMSE), and coefficient of determination (R^2^). Comparative results showed that RF outperformed MLR, GWR and SVM models. The RF gave better values of ME (0.39 cm), MAE (7.09 cm), and RMSE (10.85 cm) and higher R^2^ (62%). The sensitivity analysis demonstrated that the DEM had less uncertainty than the AL soil thickness. The outcome of the RF model indicated that elevation, flow path length and valley depth were the most important factors affecting the AL soil thickness variability across the watershed. These results demonstrated the RF model is a promising method for predicting spatial distribution of AL soil thickness using terrain parameters.

## Introduction

Active-layer (AL) soil thickness, defined as the top layer of soil, is one of the most important factors affecting soil quality and productivity, vegetation growth, soil moisture pattern, surface and subsurface flow, and shallow landslide [1–6]. Information about the spatial distribution of AL soil thickness is beneficial to hydro-ecological modeling, precision agriculture, and land resource management [7]. In most cases, the information of AL soil thickness is derived from conventional soil survey. But this method cannot offer sufficient information to satisfy the special planning (e.g. land-use planning and cropping pattern), and the application is obstructed by the high costs on time, expense and labors. Furthermore, some authors used a constant value to represent AL soil thickness across the entire landscape [8, 9] that the variability of it is ignored consequently. However, AL soil thickness is not constant over space in areas with complex terrain conditions. Therefore, alternative approaches are proposed to meet the demands of many applications.

In order to overcome the limitations of conventional soil mapping, many statistical methods have been developed for modeling the spatial pattern of soil attributes within the digital soil mapping (DSM) framework. Among them, multiple linear regression (MLR) has been widely used to quantify the soil thickness variability [5, 10–12] due to its simplicity and efficiency in computation, and easy interpretation. Ziadat established the MLR model between soil thickness and terrain variables in the Al-Muwaqqar watershed in Jordan and reported that the model was a promising approach for predicting soil attributes [11, 13]. Yang et al. investigated the relationship between soil thickness and terrain attributes using linear regression in peak-cluster depression region and showed that more than one-half of the variation in the soil thickness could be explained [12]. However, the MLR model cannot reveal the non-linear relationship between soil thickness and environmental variables that usually exists in reality [14]. Therefore, a more efficient method is required. Recently, machine learning approaches, such as support vector machines [15, 16], geographically weighted regression [17–20] and random forest [19, 20], have been applied to map soil nutrients. Previous studies have demonstrated that these methods were more intelligent due to their abilities for solving non-linearity problem [15, 16, 21]. But these applications were mostly concentrated on mapping the spatial distribution of soil organic carbon or soil organic matter.

The spatial distribution of AL soil thickness has high heterogeneity as a function of parent material, topography, climate, vegetation cover, and intense human activities [8, 17, 22–25]. Among them, topography plays a crucial role in the soil attributes variability [11, 25, 26–28], especially at a small watershed area under relatively uniform parent material, vegetation cover, and climate conditions (e.g. precipitation and temperature) [5, 29]. Topography has direct or indirect effects on the spatial distribution of soil attributes by affecting surface and underground runoff, soil erosion, soil temperature and soil formation [28], especially for slope fields with sharply rolling terrain [10, 29]. There is a strong relationship between AL soil thickness and terrain variables [5, 10, 29–32], and previous studies have demonstrated that the variables derived from digital elevation models (DEMs) could improve the prediction of AL soil thickness in different landscapes [11, 26, 33, 34]. For example Moore et al. predicted the spatial distribution of soil horizon thickness using terrain variables (e.g. slope, wetness index, and maximum flow path length), they reported that about one-half of the variation of soil attributes were explained by terrain variables [35]. Conversely, Penížek and Borůvka found the improvement of prediction models using three primary terrain variables including elevation, slope and aspect as covariates was relatively small in Southern Bohemia, they attributed this to low correlation between soil thickness and the primary variables. In the end, they conceded that the use of more terrain variables (e.g. flow path length, topographic wetness index) would provide more accurate predictions of AL soil thickness [33].

Random Forest (RF), a relatively new method, is developed from Classification and regression trees (CARTs) for improving the prediction accuracy [36]. Compared with other machine learning methods, RF has several advantages, such as resistance to overfitting, insensitive to noise features, robust error estimation, simplicity in parameters defined, and quantifying variable importance [20, 36, 37]. In this paper, the overall purpose was to predict the spatial pattern of AL soil thickness based on terrain variables at a small watershed scale using RF model. More specifically, the objectives of this study were to (1) investigate the capability of RF model for predicting the AL soil thickness by comparing it with MLR, GWR and SVM based on a validation set, (2) estimate uncertainty using Monte Carlo simulation method, (3) model the spatial distribution of AL soil thickness, and (4) explore the main factors affecting the AL soil thickness variability.

## Materials and methods

### Study area

**Fig 1:**
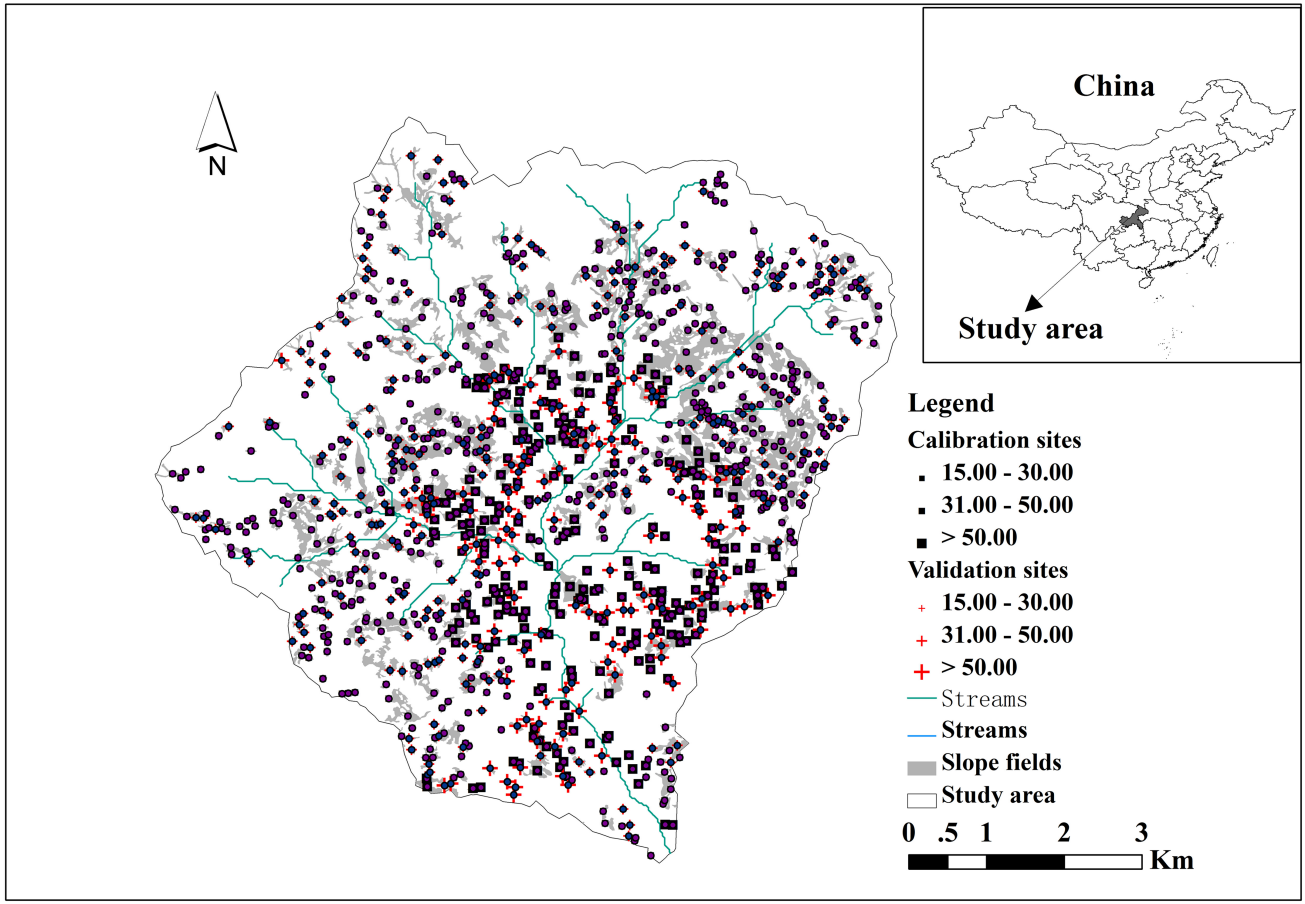
Maps of the watershed location and soil sampling sites.

The study site (31°6’26”– 31°11’41” N, 109°35’9”– 109°41’11” E) covering 5376.374 ha is located within a small watershed area of Fengjie County of Chongqing, southwest China (Fig 1). The topography of the study area is mainly mountainous with elevation varying between 238 and 1592 m above sea level. The climate is moderate subtropical with vertical characteristic. The average annual precipitation is 1132 mm which mainly happens in summer and the mean annual temperature decreases along with the increasing of elevation(16.4°C, 13.7–16.4°C, and < 10.8°C for areas below 600 m, within the range 1000–1400 m, and above 1400 m, respectively). The average annual sunlight length of the area is 1639 h. The predominant parent materials are feldspar-quartz sandstone, limestone and dolomite. The soil types consist of yellow earths, purplish soils, and limestone soils [38]. The dominant cropping system is wheat-maize rotation across the study site. The soil is neutral with a pH value of 7.2 and content of organic matter is 17.4 g/kg for the watershed.

### Soil data

A total of 1113 soil samples were collected from slope fields over the watershed using a manual auger in October of 2007 when crops were harvested. The study did not involve private land, protected land, endangered or protected species. No specific permissions were required for these locations/activities. The position and elevation of each soil sample were georeferenced using a global positioning system (GPS), and other relevant information such as cropping system was recorded at the same time. AL soil thickness was determined by digging soil profiles and measuring the depth to the non-soil portion of the soil profile. The samples were randomly divided into calibration (770 soil samples) and validation (343 soil samples) sets. The calibration set was used to develop models for predicting AL soil thickness and the validation set was used to evaluate the performance of the models.

### Terrain variables

**Fig 2:**
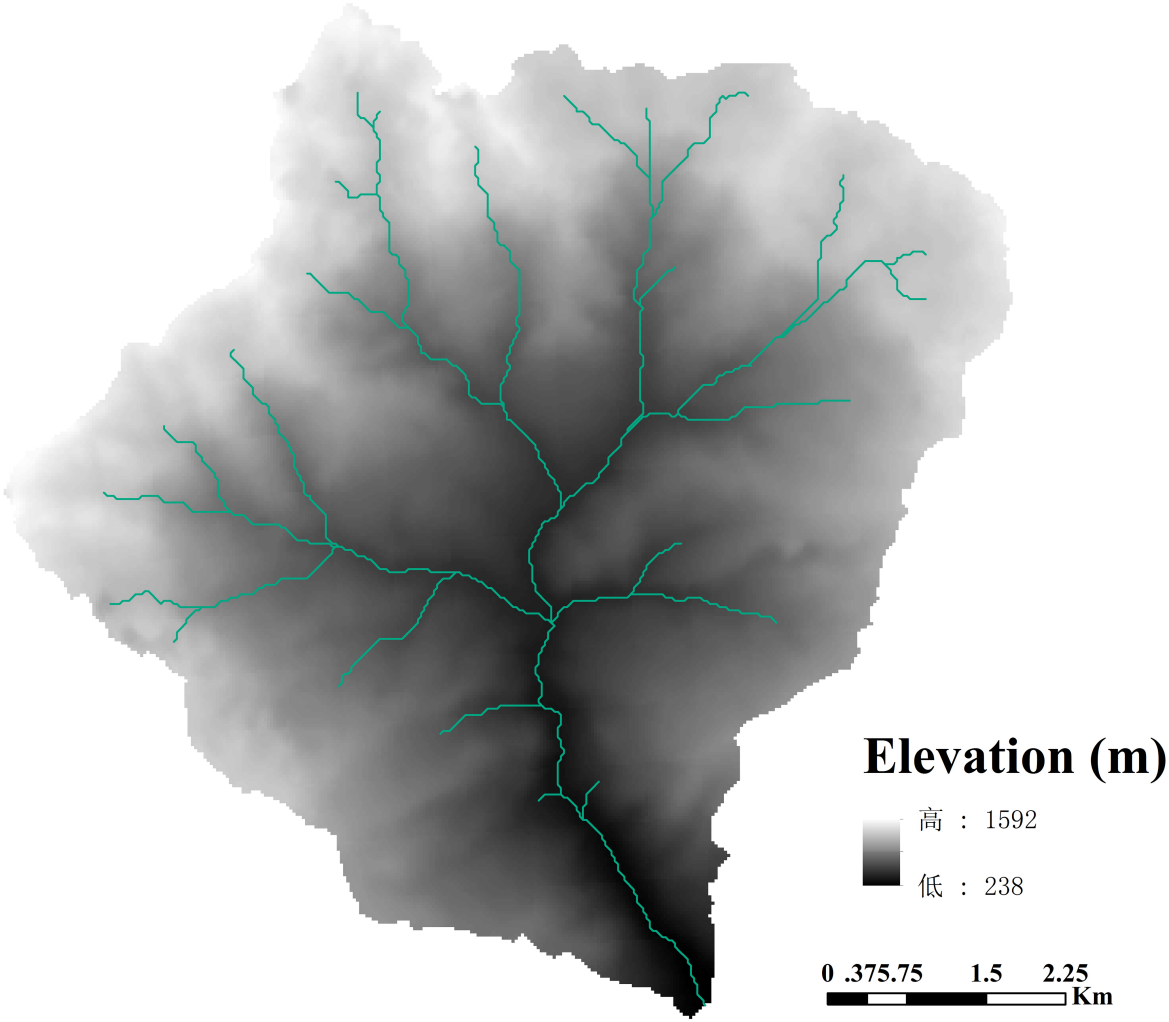
The digital elevation model of the study area.

ASTER GDEM (Global Digital Elevation Model) was used in this study (Fig 2). It produced by NASA and METI of Japan and can be obtained from Geospatial Data Cloud hub published by Chinese Academy of Sciences (2009). Pre-production estimated accuracies for this product are 20 meters at 95% confidence for vertical data and 30 meters at 95% confidence for horizontal data. The coordinate system of the data is WGS84 and the original data is in IMG format. Seven terrain variables, namely, elevation, aspect, relative slope position (RSP), valley depth (VD, m), flow path length (FPL, m), topographic wetness index (TWI), and slope height (SH, m), were derived from the DEM (S1 Table) using SAGA GIS software [39]. In order to determine an appropriate prediction model in this study, all seven terrain variables will be used for these models.

### Multiple linear regression model

As one of the most widely used regression methods, multiple linear regression model (MLR) based on ordinary least square method was adopted to predict the AL soil thickness spatial distribution in this study. The MLR model is expressed as follow:

$${\text{Y}}_{i}=\beta_{0}+\beta_{1}{\text{X}}_{\text{i}1}+\beta_{2}{\text{X}}_{\text{i}2}+\beta_{3}{\text{X}}_{\text{i}3}\ldots +\beta_{\text{n}}{\text{X}}_{\text{in}}$$ (1)

where *Y*_*i*_ (*i = 1*,*2*,*3*,*…*,*n*) and *β*_*j*_(*j = 0*,*1*,*2*,*…n*) are the dependent variable and regression coefficient, respectively. *X*_*i*_ (*i = 1*,*2*,*3*,*…*,*n*) is the independent variable. The model will be built based on the linear relationship existing between dependent and independent variables. Moreover, input and output data must be numeric. In the current study, the selected terrain variables were independent variables and the AL soil thickness was dependent variable.

### Geographically weighted regression

Geographically weighted regression (GWR) is an exploratory technique that mainly intends to indicate where non-stationarity is taking place on the map [17], that is, where locally weighted regression coefficients move away from their global values. The technique is fully described by Fotheringham et al. (2002) and involves first selecting a bandwidth for an isotropic spatial weights kernel, typically a Gaussian kernel with a fixed bandwidth chosen by leave-one-out cross-validation. A standard GWR can be defined as follow:

$${\text{Y}}_{i}=\beta_{0}(u_{i},v_{i})+\beta_{1}(u_{i},v_{i}){\text{X}}_{\text{i}1}+\beta_{2}(u_{i},v_{i}){\text{X}}_{\text{i}2}+\ldots +\beta_{\text{n}}\left(u_{i},v_{i}\right){\text{X}}_{\text{in}}$$ (2)

where *Y*_*i*_ (*i = 1*,*2*,*3*,*…*,*n*) is the predicted AL soil thickness at the *i*th location; (*u*_*i*_,*v*_*i*_) is the coordinate at the *i*th location; and *β*_*n*_(*n = 0*,*1*,*2*,*…n*) is the regression coefficient; X_in_ (*in = i1*,*i2*,*…*,*in*) is environmental variable for the *i*th location; and *n* is the number of environment variables.

### Support vector machine

Support vector machine (SVM), a machine learning algorithm, is based on statistical theory and structural risk minimization principle. SVMs map the sample space into a high-dimensional or infinite-dimensional feature space based on Mercer’s nuclear [40]. Then, the highly non-linear problem of sample space could be solved using linear learning technique in the feature space [41]. The function (f(x)) for non-linear regression is the extension of linear regression by introducing a kernel function.

Given a set of training data, T = {(x_i_,y_i_), i = 1, 2, … n}, where x_i_ and y_i_ are the input and output values of the ith data, respectively, the f(x) is defined as an approximately linear regression problem and expressed as,

f(x)=ω⋅φ(x)+b (3)

where φ(x) is the mapping function, ω is an adjustable weight vector, b is the scalar threshold.

In order to avoid the generation of dimension disaster problem during the computation in feature space and solve the non-linear problem, a kernel function is introduced. In the current study, a radial basis function (Eq 4) which satisfied the Mercer’s condition was used as the kernel function.

$$\text{K}\left({\text{x},\text{x}}_{\text{i}}\right){=\text{exp}}^{-{\left({2\sigma }^{2}\right)}^{-1}{\left|\left|{\text{x}-\text{x}}_{\text{i}}\right|\right|}^{2}}$$ (4)

where σ is the bandwidth parameter. After introducing the kernel function, the sample data can be converted into linear separable data in a high-dimensional feature space. Support vector regression is a kind of regression algorithm based on penalty learning [37]. An ε-insensitive loss function [38] is introduced in SVM and the formula is,

$${\text{l}}_{\varepsilon }={\left|\text{y}-\text{f}\left(\text{x}\right)\right|}_{\varepsilon }=\{\begin{array}{ll} 0 & \text{if }\left|\text{y-f}\left(\text{x}\right)\right|\leq \varepsilon \\ \left|\text{y}-\text{f}\left(\text{x}\right)\right|-\varepsilon & \text{otherwise} \end{array}$$ (5)

where ε is the insensitive coefficient, which is used to control the accuracy of the prediction. Based on the structural risk minimization, the regression problem can be transformed to an optimization problem by the following way.

$$\text{R}\left(\text{C}\right){=\text{Cn}}^{-1}\sum_{\text{i}=1}^{\text{n}}l_{\varepsilon }{+2}^{-1}\left\|\omega \right\|$$ (6)

where C is the penalty weight, which controls the extent of penalty on the samples (which their prediction errors are beyond the range of ε) and realizes the trade-off between the number of samples and the model complexity. In generally, the value of C should not be too large in order to avoid overlearning problem. In view of the existence of samples with prediction errors are larger than the non-negative constant ε, the non-negative slack variables (ξ_i_, ξ_i_*) are introduced. Therefore, the optimization function for computing the empirical risk minimization problem is equivalent to solve the following constrained optimization problem.

$$\text{Min}.\text{ R}\left({\omega ,\xi }_{\text{i}}{,\xi }_{\text{i}}{}^{*}\right)=2^{-1}{\left|\left|\omega \right|\right|}^{2}+\text{C}\overset{\text{n}}{\underset{\text{i}=1}{\sum }}\left(\xi_{\text{i}}{+\xi }_{\text{i}}{}^{*}\right)$$

$$\text{Subjected to}\{\begin{array}{c} {\text{y}}_{\text{i}}-\text{f}\left({\text{x}}_{\text{i}}\right)\leq {\varepsilon +\xi }_{\text{i}}\\ \text{f}\left({\text{x}}_{\text{i}}\right){-\text{y}}_{\text{i}}\leq {\varepsilon +\xi }_{\text{i}}{}^{*} \end{array}\text{i}=1,2,3,\ldots n$$ (7)

In fact, the above Eq (7) is a typical quadratic programming problem. According to the dual theory, Lagrangian function (a_i_, a_i_*) and kernel function (K(x, x_i_)) are introduced to transformthe above problem into a dual optimization problem. The dual form can be expressed as,

$$\text{Q}({\text{a}}_{\text{i}},{\text{a}}_{\text{i}}{}^{*})=\sum_{\text{i}=0}^{\text{n}}{\text{y}}_{\text{i}}\left({\text{a}}_{\text{i}}+{\text{a}}_{\text{i}}{}^{*}\right)-2^{-1}\sum_{\text{i}=0}^{\text{n}}\sum_{\text{j}=0}^{\text{n}}\left({\text{a}}_{\text{i}}-{\text{a}}_{\text{i}}{}^{*}\right)\left({\text{a}}_{\text{i}}-{\text{a}}_{\text{i}}{}^{*}\right)\text{K}\left({\text{x}}_{\text{i}},{\text{x}}_{\text{j}}\right)-\varepsilon \sum_{\text{i}=0}^{\text{n}}\left({\text{a}}_{\text{i}}+{\text{a}}_{\text{i}}{}^{*}\right)$$

$$\text{Subjected to}\{\begin{array}{c} \sum_{\text{i}=1}^{\text{n}}\left({\text{a}}_{\text{i}}{-\text{a}}_{\text{i}}{}^{*}\right)=0\\ 0\leq {\text{a}}_{\text{i}}{,\text{a}}_{\text{i}}{}^{*}\leq \text{C} \end{array}\text{i}=1,2,3\ldots \text{n}$$ (8)

where a_i_ and a* are Lagrange multipliers. Support vectors consist of the sample points which satisfy the equality of a_i_−a*≠ 0. Therefore, the optimization hyperplane linear regression function can be written as,

$$\text{f}\left(\text{x}\right)=\sum_{\text{i}=1}^{\text{n}}\left({\text{a}}_{\text{i}}{-\text{a}}_{\text{i}}{}^{*}\right)\text{K}\left({\text{x},\text{x}}_{\text{i}}\right)+\text{b}$$ (9)

In this study, the parameters C and σ were set to be 0.87 and 13 reckoned by Grid search approach, respectively. The SVM model was calculated in Matlab7.12.0.

### Random forest

Random forest (RF) is a forest which combines multiple unrelated classification or regression trees (CARTs) [34]. Bootstrap sampling (with replacement) is used to generate a random sample from original dataset for each tree. Each tree is built based on a random subset which is selected from training dataset by bootstrap sampling (with replacement). The number of trees (n_tree_) defined by users is identical to the random subsets. The trees are grown by binary recursive partitioning [42], the output variable at each tree is partitioned into multiple binary subsets (each subset with maximum homogeneity). For each node, a random subset selected from the corresponding input variables is used. The best variable is searched for each binary split by the least impurity of node. The size of each randomly selected subset of input variables (m_try_) remains unchanged within the whole forest growing procedure and needs specification. Furthermore, the bias of each tree can be reduced as it is grown without pruning [36]. RF can be used to establish classification and regression model. The final output in classification model is a majority vote of the outputs of the trees. However, in regression model, the prediction value is an average of the outputs of all trees and the formula is expressed as,

h¯(x)=(1k)∑i=1kh(X;θi) (10)

where $\bar{h}\left(x\right)$ is a prediction value, *θ*_*i*_ is a random vector with independent and identically distributed, *X* is an input matrix, *h(X; θ*_*i*_*)* is an output of a tree, *k* is the number of trees. In regression model, three parameters (n_tree_: the number of trees, nodesize: the minimum size of observations at each terminal node, m_try_: the number of input variables at each node) are defined by users. Theoretically, the prediction accuracy increases as the number of trees increases. However, the computational load increases and the speed of performance improvement decreases with the increasing of the forest size. As a randomly selected subset of input variables is used for splitting at each node, the correlation between the trees is decreased, the calculation time is reduced and the performance of the forest is improved.

Each tree is trained on about two-third of the training data and tested on the reminder which is called out-of-bag (OOB) samples. Then, the out-of-bag error (OOB error) is estimated [36]. The OOB error is an unbiased estimator and is identical with the prediction error which is estimated by a test set with the same size of the random subset [36]. The mean square error (MSE_OOB_) of the forest is calculated by aggregating the OOB predictions and the expression is given as,

MSEOOB=1n∑i=1n(zi−z^iOOB)2 (11)

where z_i_ is the OOB prediction of the *i*th point, ${\hat{z}}_{i_{\text{OOB}}}$ is the average of OOB predictions. Moreover, the variable importance also can be assessed based on the OOB error. The increased in MSE_OOB_ (InMSE) is used to quantify the importance of variable. The variable with a higher value of InMSE was more important than others.

For the RF model we used the “random Forest” package [43] in R [44]. After several experiments, n_tree_, nodesize, and m_try_ were set to be 2000, 3, and 5, respectively, in this study.

### Accuracy assessment

In order to assess the performance of models for AL soil thickness mapping, mean error (ME), mean absolute error (MAE), root mean squared error (RMSE), and determination coefficient (R^2^) were calculated by the measured and predicted values from the validation set. The formulas were defined as follows.

ME=1n∑i=1n(Ai−Bi) (12)

MAE=1n∑i=1n|Ai−Bi| (13)

RMSE=1n∑i=1n(Ai−Bi)2 (14)

$${\text{R}}^{2}=\frac{\sum_{\text{i}=1}^{\text{n}}{\left({\text{A}}_{\text{i}}-\bar{\text{A}}\right)}^{2}{\left({\text{B}}_{\text{i}}-\bar{\text{B}}\right)}^{2}}{\sum_{\text{i}=1}^{\text{n}}{\left({\text{A}}_{\text{i}}-\bar{\text{A}}\right)}^{2}\sum_{\text{i}=1}^{\text{n}}{\left({\text{B}}_{\text{i}}-\bar{\text{B}}\right)}^{2}}$$ (15)

where n is the number of validation sites, *A*_*i*_ and *B*_*i*_ represent the measured and predicted values of AL soil thickness, respectively. $\bar{\text{A}}$ and $\bar{\text{B}}$ are mean values of the measured and predicted AL soil thickness, respectively. Models with lower values of ME, MAE, RMSE and a higher value of R^2^ perform better.

### Statistical analysis

Descriptive statistical analyses of minimum, maximum, mean, standard deviation (SD), and coefficient of variation (CV) were applied to test the variation of AL soil thickness and terrain variables. Correlation analysis was used to find the relationship between AL soil thickness and terrain variables. All of the statistical analysis and the multiple linear regression were computed using SPSS 19.0 software.

### Uncertainty analysis

Uncertainties within the best performed model, which were caused by terrain variables and collected AL soil thickness data, were analyzed through Monte Carlo method [18]. A number of input and output variables were generated based on their probability distribution functions. In this study, 1000 estimates of DEM and AL soil thickness were generated. The seven terrain indicators were derived from the created DEMs. The uncertainties in DEM and AL soil thickness were then estimated using the best performed model. This analysis was conducted by using R [44] and Feature Manipulate Engine 2015 (FME) software.

## Results

### Descriptive statistical analysis

The descriptive statistics of AL soil thickness and terrain variables were summarized in (Table 1). The AL soil thickness values of all sampling sites in the study area varied from 15 to 60 cm with a mean of 35.39 cm and standard deviation (SD) of 17.495 cm. The coefficient of variation (CV) was used to estimate the variability of AL soil thickness. The CV for AL soil thickness was 49.44%, which indicated that AL soil thickness had intermediate variability. For terrain variables, all had intermediate variability with CV varying between 28.82% and 84.46%. It seems that the variability of AL soil thickness might be controlled by the complexity of topography of the study area. The statistical results of AL soil thickness for both calibration and validation sets were very similar to that of all sampling data, which indicated the small samples were suitable for developing and verifying the prediction models in this work.

**Table 1:** Descriptive statistics of AL soil thickness and terrain variables.

Variable	N	Min.	Max.	Mean	SD	CV(%)
Elevation (m)	1113	249	1546	891.55	297.21	33.34
Aspect (°)	1113	2.12	360	174.2	94.37	54.17
RSP	1113	0	1	0.43	0.325	75.58
VD (m)	1113	0	465	143	107.03	74.61
FPL (m)	1113	0	3889	1207	770.26	63.80
TWI	1113	3.76	21.82	6.45	1.86	28.82
SH (m)	1113	2.76	4596	94	79.58	84.46
ST: All sampling sites (cm)	1113	15	60	35.39	17.495	49.44
ST: Calibration sites (cm)	770	15	60	35.47	17.487	49.30
ST: Validation sites (cm)	343	15	60	35.2	17.537	49.82

### Correlation analysis

The relationships between AL soil thickness and terrain variables were explored by correlation analysis (S2 Table). As the table shown, there were significant correlations between AL soil thickness and terrain variables in this study site (Table 2). AL soil thickness was negatively correlated with elevation (r = -0.510, p < 0.01), RSP (r = -0.207, p < 0.01) and SH (r = -0.098, p < 0.01), and positively correlated with aspect (r = 0.089, p < 0.01), VD (r = 0.225, p < 0.01), FPL (r = 0.272, p < 0.01), TWI (r = 0.061, p < 0.05). This implied that elevation had higher impacts on the AL soil thickness variability, whereas TWI had little impacts on the AL soil thickness variability. The results suggested that topography had strong effects on AL soil thickness.

**Table 2:** Relationships between AL soil thickness and terrain variables.

	Elevation	Aspect	RSP	VD	FPL	TWI	SH
ST	-0.510**	0.089**	-0.207**	0.225**	0.272**	0.061*	-0.098**

* and ** denote significance levels at p < 0.05 and p < 0.01, respectively.

### Model performance

In order to quantify the performance of models, the accuracy indicators (mean error, mean absolute error, root mean squared error, and R^2^) were calculated using the validation data set (343 soil samples) (S3 Table) and the results were listed in (Table 3). The mean error (ME) was calculated to identify the bias of predictions. The ME of MLR, SVM, GWR and RF were -0.32, 1.42, 0.01, and 0.39 cm, respectively. The negative value suggested MLR model underestimated the AL soil thickness values, while the positive value revealed overestimation of SVM, GWR and RF models. The extent of AL soil thickness estimated by RF model was much narrow than that of SVM. Furthermore, the MAE (7.09 cm) and RMSE (10.85 cm) of RF model were lower than that of MLR and SVM models, and the R^2^ of RF (62%) was much higher than that of MLR (27%) and SVM (49%) models. In terms of RMSE and R^2^, RF model outperformed others for predicting the spatial distributions of AL soil thickness in this study area.

**Table 3:** Accuracy assessment indices of different methods based on validation set.

	ME(cm)	MAE (cm)	RMSE (cm)	R^2^
MLR	-0.32	12.83	14.95	0.27
GWR	0.01	6.23	11.48	0.59
SVM	1.42	8.57	12.67	0.49
RF	0.39	7.09	10.85	0.62

**Fig 3:**
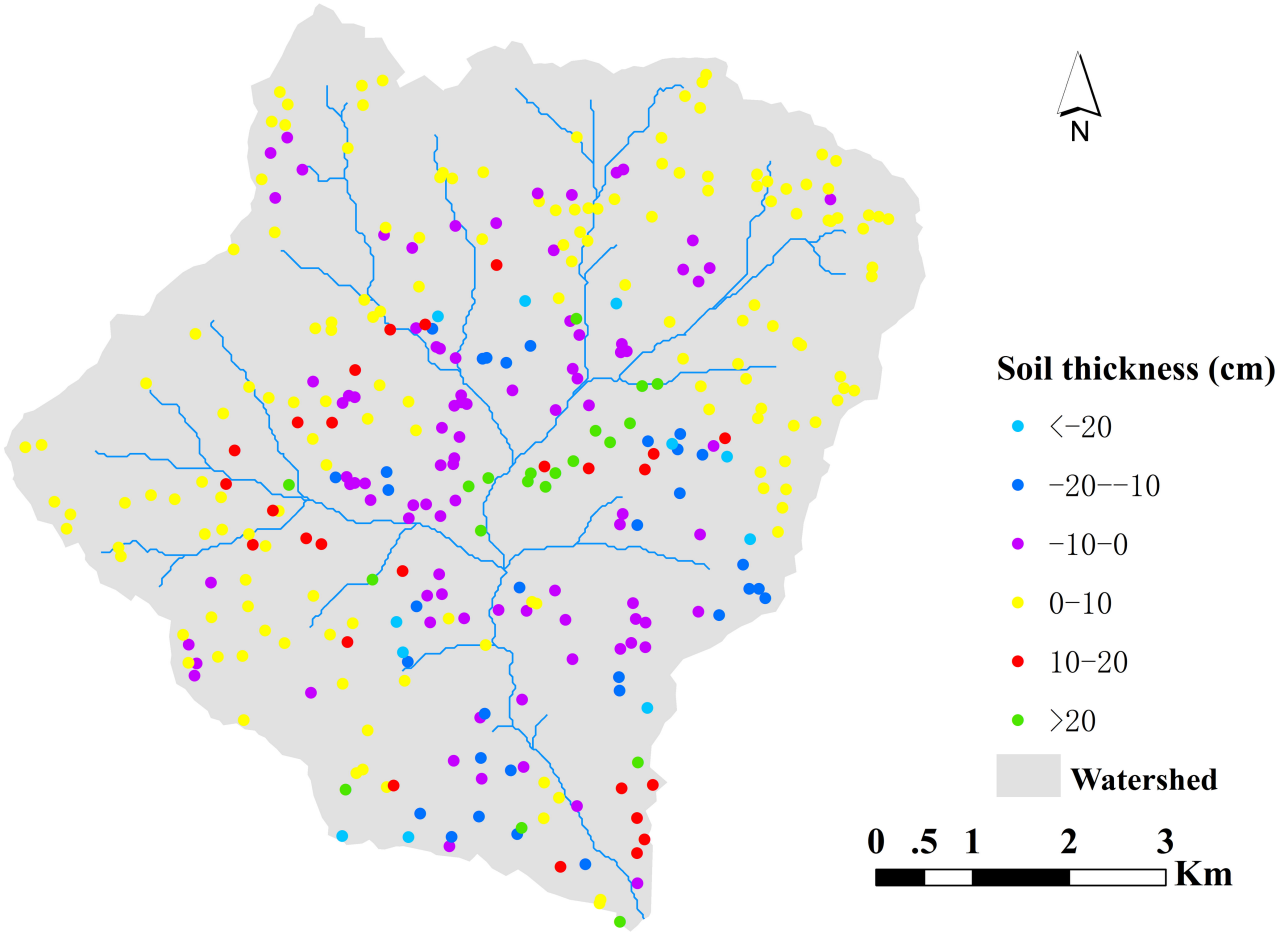
Spatial distribution maps of prediction deviation of AL soil thickness produced by RF.

ME, mean error; MAE, mean absolute error; RMSE, root mean square error; R^2^, coefficient of determination.

(Fig 3) showed that bigger errors were mostly found in the central areas with longer flow path length. The uncertainties in input and output variables were estimated and the contributions of them to the best performed model (RF) were determined (S4 Table). The results were given in (Table 4). The changes of RMSE and R^2^ indicated that the uncertainty in AL soil thickness was bigger than that of DEM.

**Table 4:** Contributions of uncertainties in DEM and AL soil thickness to RF model.

	DEM	AL Soil thickness
RMSE	41.4%	58.5%
R^2^	45.2%	54.8%

### Spatial distribution of AL soil thickness

**Fig 4:**
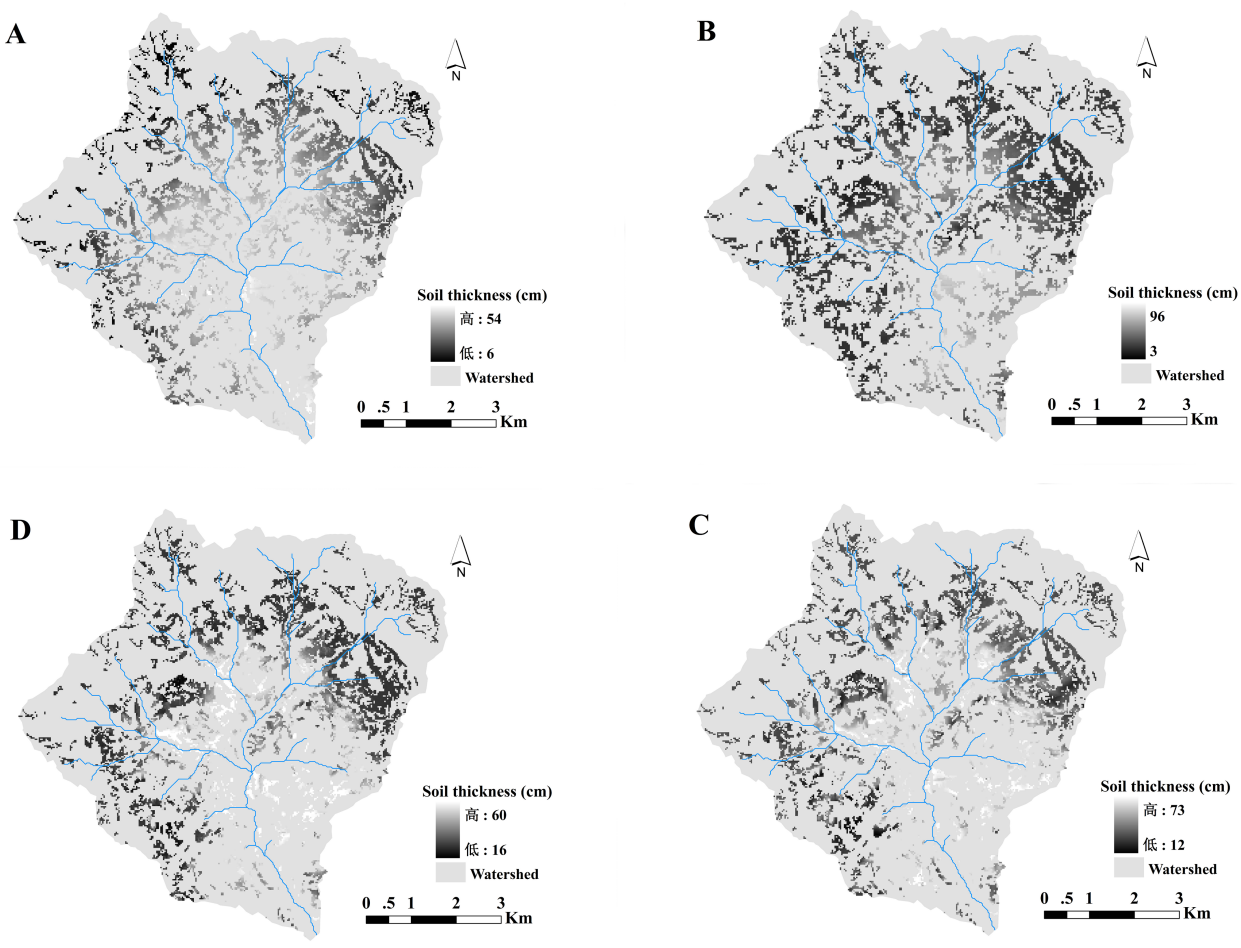
Spatial distribution maps of AL soil thickness produced by (A) MLR, (B) GWR, (C) SVM, and (D) RF.

The spatial distribution maps of AL soil thickness produced by MLR, SVM, GWR and RF models were shown in (Fig 4). The four models produced similar distributional trends of AL soil thickness over the study area. Higher values of AL soil thickness mainly concentrated in valley bottoms and lower areas with low elevation, whereas lower values of AL soil thickness mainly located around of valley with high elevation. These results suggested that elevation was the notable variable affecting the spatial distribution of AL soil thickness across the watershed. The spatial distribution map of AL soil thickness produced by MLR and GWR reflected more smooth spatial variation and fewer details in spatial variation of AL soil thickness in the local area. On the contrary, the distribution maps predicted by SVM and RF showed more particular spatial variation information such as in the valley bottoms where the predicted values by SVM and RF were higher than those by MLR and GWR. Moreover, the range of AL soil thickness values produced by RF model was closer to the measured values than those predicted by MLR, SVM and GWR.

### Variable importance

**Fig 5:**
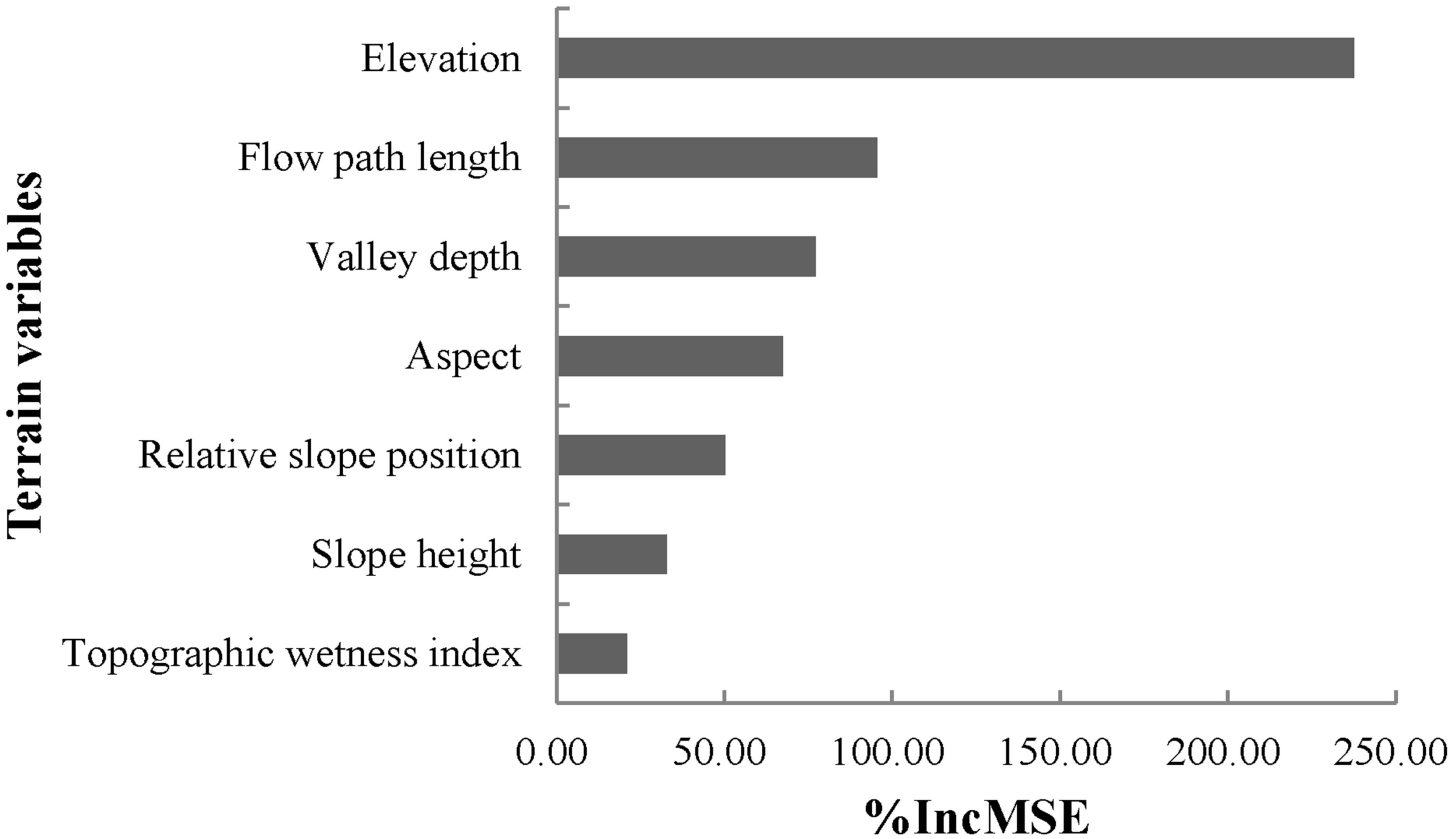
Variable importance produced by RF model.

The relative importance of terrain variables yielded by the best regression model (RF) was shown in (Fig 5). The terrain variables were ranked in order of elevation > flow path length > valley depth > aspect > relative slope position > slope height > topographic wetness index. Among these terrain variables, elevation with InMSE of 237.59% was the most critical variable affecting the variation of AL soil thickness in the current area. Flow path length and valley depth with InMSEs of more than 75% were the second and the third most important variables controlling AL soil thickness variability, respectively.

## Discussion

The comparison of the accuracy indictors (ME, MAE, RMSE and R^2^) suggested that RF was the best method for modeling the spatial distribution of AL soil thickness. RF model outperformed others for predicting AL soil thickness benefited from its insensitivity to over-fitting and avoid noise and its predominance in dealing with the non-linear problem [19, 36]. Lark indicated that the complex non-linear relationships between soil attributes and terrain variables are subsistence, a simple linear model cannot catch these relationships [45]. This could explain why the MLR and GWR model gave worse performance in this study area. Although the SVM algorithm is able to catch the non-linear relationships, its performance is still subject to certain restrictions, such as the difficulty in finding the suitable kernel function and optimal parameters. Furthermore, the range of AL soil thickness estimated by RF was closer to the measurements than that of SVM (Fig 3), this might because RF performed better on avoiding the overfitting problem which involves high-dimension explanatory variables [46]. Additionally, RF algorithm also has other advantages over SVM, GWR and MLR approaches. For instance, RF does not need to preprocess the raw data before building the prediction model, and the importance of explanatory variables can be measured using the OOB dataset [19].

The spatial distribution maps of AL soil thickness produced by MLR, GWR, SVM and RF showed a similar spatial pattern trend over the watershed, where much thicker soils mostly concentrated in valley and lower areas while lower ones mainly occurred in steep slopes and higher areas. This was also found in other landscape. For example, Mehnatkesh et al. made a study in the semiarid hilly region in western Iran [5]. They reported that higher values of AL soil thickness existed in the footslope and toeslope. Scarpone et al. also found a similar distribution trend in AL soil thickness in southern British Columbia [47]. A possible explanation for this might be the effects of complex topography, since topography indirectly influence the spatial distribution of AL soil thickness by affecting surface runoff, soil erosion, soil deposition and soil temperature, especially in slope fields with sharply rolling terrain. Thus, the spatial heterogeneity of AL soil thickness is a result of the interaction of soil process (soil erosion and soil deposition) and surface runoff. In the current study, elevation which showed highly negative correlation with AL soil thickness was the most important one affecting the spatial pattern of AL soil thickness over the watershed. Soil erosion could be accelerated under the increases of elevation in hilly slopes especially around the hilltop, while soil deposition is intensified in valley bottoms and lower areas [47]. Under the interaction of soil erosion and soil deposition, therefore, the valley bottoms with relatively lower elevation had thicker soils, and around of valley with higher elevation had shallower soils. In addition, the relative importance of terrain variables also showed that flow path length and valley depth were the second and the third most important factors, respectively, which both showed highly positive correlation with AL soil thickness. The values of AL soil thickness in this watershed increased with the increases of flow path length and valley depth. Generally, surface water always accumulated in concave areas and valley with high values of valley depth [10, 30, 48], where AL soil thickness is thicker resulting from fluvial deposition processes [47]. The flow path length can reflect the spatial pattern of runoff, and deeper surface water can be observed in the area with relative longer flow path length [49]. Hence, thicker soils concentrated in the concave areas and valley with longer flow path length and higher valley depth.

The uncertainty of the best performed model could be attributed to both DEM and AL soil thickness. Many studies found that some subtle details of the terrain features become less discernible and the predictive efficiency of terrain variables is lost rapidly with a coarse resolution of DEM [50, 51]. Hence, the accuracy of terrain variables could be affected by the resolution of DEM [27, 52, 53], such as smaller mean slope and higher mean wetness index are produced as the decrease of the resolution. This could reduce the predictive capability of the models for the spatial distribution of AL soil thickness [12]. Additionally, the consideration of objects on the earth surface of DEMs also yields uncertainty in digital terrain, which could further impact on models’ performance. However, this will not affect the results of the current work which was conducted at a small watershed of a rural area. Moreover, the outcomes of the sensitivity analysis suggested that the uncertainty in AL soil thickness was bigger than that of DEM. During the work, the sample locations were identified by the local trained experts and the digging depths were mainly determined by their subjective experiences.

## Conclusion

In this study, we are devoted to find an acceptable, simple, effective, and low cost soil-landscape modeling approach using limited observations to predict the spatial pattern of AL soil thickness at watershed scale. Seven quantitative terrain indexes including elevation, aspect, relative slope position, valley depth, flow path length, slope height, and topographic wetness derived from DEM were applied with MLR, SVM, and RF methods. The spatial distribution maps of AL soil thickness produced by all models illustrated that thicker soils mostly concentrated in valley bottoms and lower areas and shallower soils mainly occurred in steep slopes and higher areas. According to the performance indicators, RF model which could simulate about 62% of the AL soil thickness variability outperformed others in the study area. Because the RF model can not only solute the non-linear problems between AL soil thickness and terrain variables but also avoid overfitting phenomenon and resist noise in predictors. The importance of variables calculated by RF indicated that elevation, flow path length, and valley depth were the most important terrain variables affecting the AL soil thickness variability across the watershed. These variables indirectly affect AL soil thickness mainly by controlling soil erosional and depositional processes.

The outcomes of the current study suggested that RF technique is a useful tool for generating the digital AL soil thickness map at watershed scale. Of course, the generality and transportability of the RF model to other landscapes with similar features as that in the current area still remains to be verified. Furthermore, a high-quality digital terrain model (DTM) is needed to find an optimum resolution for improving the accuracy of predictive model.

## Supporting information

S1 Table Statistical analysis of AL soil thickness and terrain variables. (XLSX)

S2 Table Relationships between AL soil thickness and terrain variables. Significance levels can be judged by Signif.code.
(XLSX)

S3 Table Accuracy assessment indices of four methods: ME, MAE, RMSE and R^2^. (XLSX)

S4 Table Uncertainties assessment in DEM and AL soil thickness to RF model. (XLSX)
